# Differential Pain‐Related Behaviors and Bone Disease in Immunocompetent Mouse Models of Myeloma

**DOI:** 10.1002/jbm4.10252

**Published:** 2019-12-30

**Authors:** Marta Diaz‐delCastillo, Danna Kamstrup, Rikke Brix Olsen, Rie Bager Hansen, Thomas Pembridge, Brigita Simanskaite, Juan Miguel Jimenez‐Andrade, Michelle A Lawson, Anne‐Marie Heegaard

**Affiliations:** ^1^ Department of Drug Design and Pharmacology, Faculty of Health and Medical Sciences University of Copenhagen Copenhagen Denmark; ^2^ Department of Oncology & Metabolism University of Sheffield Sheffield UK; ^3^ Mellanby Centre for Bone Research University of Sheffield Sheffield UK; ^4^ Department of Unidad Académica Multidisciplinaria Reynosa Aztlan Universidad Autónoma de Tamaulipas Reynosa, Tamaulipas Mexico

**Keywords:** BONE QCT/microCT, BONE–BRAIN–NERVOUS SYSTEM INTERACTIONS, PRECLINICAL STUDIES, TUMOR‐INDUCED BONE DISEASE

## Abstract

Bone pain is a serious and debilitating symptom of multiple myeloma (MM) that impairs the quality of life of patients. The underlying mechanisms of the pain are unknown and understudied, and there is a need for immunocompetent preclinical models of myeloma‐induced bone pain. The aim of this study was to provide the first in‐depth behavioral characterization of an immunocompetent mouse model of MM presenting the clinical disease features: osteolytic bone disease and bone pain. We hypothesized that a widely used syngeneic model of MM, established by systemic inoculation of green fluorescent protein‐tagged myeloma cells (5TGM1‐GFP) in immunocompetent C57Bl/KaLwRijHsd (BKAL) mice, would present pain‐related behaviors. Disease phenotype was confirmed by splenomegaly, high serum paraprotein, and tumor infiltration in the bone marrow of the hind limbs; however, myeloma‐bearing mice did not present pain‐related behaviors or substantial bone disease. Thus, we investigated an alternative model in which 5TGM1‐GFP cells were directly inoculated into the intrafemoral medullary cavity. This localized myeloma model presented the hallmarks of the disease, including high serum paraprotein, tumor growth, and osteolytic bone lesions. Compared with control mice, myeloma‐bearing mice presented myeloma‐induced pain‐related behaviors, a phenotype that was reversed by systemic morphine treatment. Micro‐computed tomography analyses of the myeloma‐inoculated femurs showed bone disease in cortical and trabecular bone. Repeated systemic bisphosphonate treatment induced an amelioration of the nociceptive phenotype, but did not completely reverse it. Furthermore, intrafemorally injected mice presented a profound denervation of the myeloma‐bearing bones, a previously unknown feature of the disease. This study reports the intrafemoral inoculation of 5TGM1‐GFP cells as a robust immunocompetent model of myeloma‐induced bone pain, with consistent bone loss. Moreover, the data suggest that myeloma‐induced bone pain is caused by a combinatorial mechanism including osteolysis and bone marrow denervation. © 2019 The Authors. *JBMR Plus* published by Wiley Periodicals, Inc. on behalf of American Society for Bone and Mineral Research.

## Introduction

Multiple myeloma (MM) is a largely incurable neoplasia of plasma cells with an incidence of six people in a population of 100,000 per year in Europe.^1^ Approximately 90% of MM patients develop bone disease,^2^ which is characterized by an imbalance in the bone remodeling process that leads to the development of purely osteolytic lesions. Often these lesions induce hypercalcemia,^3^ pathological fractures,^4^ and spinal cord compression,^5^ and are associated with decreased survival expectancy.^6^

The commonest symptom of MM is bone pain, which affects 70% of myeloma patients,^7^, ^8^, ^9^, ^10^ and up to 90% in the advanced stages of the disease.^11^ Myeloma‐induced bone pain is often described as severe, and it impairs the patient's quality of life.^12^ As the survival expectancy has been reported to increase thanks to the development of new therapies for the disease treatment,^13^ novel analgesic avenues for the treatment of bone pain in myeloma patients are called for. Surprisingly, little is known about the characteristics and underlying mechanisms of myeloma‐induced bone pain.

Although several preclinical models of MM have been developed over the years with the goal of understanding disease etiology and identifying novel drug targets, only one xenograft mouse model of MM has been evaluated in terms of myeloma‐induced bone pain.^14^ Xenograft models, however, rely on the use of immunosuppressed mice, whereas MM is a plasma cell cancer and mounting evidence suggests that the immune system plays a pivotal role in chronic pain development.^15^, ^16^, ^17^, ^18^ Thus, there is a clear need for a superior model of myeloma‐induced bone pain that integrates the immune component.

To address this, we studied pain‐related behaviors in a commonly used syngeneic model of MM, in which a subclone of myeloma cells (5TGM1) is propagated by tail vein injection in C57BL/KaLwRijHsd (BKAL) mice.^19^ We found that these mice display a phenotype consistent with MM, including high serum paraprotein, tumor growth in the bone marrow, and splenomegaly,^19^, ^20^ but did not present bone loss or a pain‐like phenotype. Consequently, we investigated pain‐related behaviors in a variant of this model in which 5TGM1 cells are intrafemorally inoculated, and show that these mice present high serum paraprotein, pain‐like behaviors, significant bone destruction, and alterations in the distribution of sympathetic and sensory neurons innervating the hind limb bones. This localized, syngeneic model of MM is a powerful and clinically relevant tool to understand myeloma‐induced bone pain.

## Materials and Methods

### Cell culture

Green fluorescent protein‐ (GFP‐) tagged 5TGM1 cells^21^ were maintained by serial passage and cultured in vitro for less than 10 days^22^, ^23^ in Roswell Park Memorial Institute media with glutamine and phenol red, supplemented with 10% FBS, 1% penicillin–streptomycin (100 U/100 μg/mL), 1% sodium pyruvate (1mM), and 1% MEM nonessential amino acids (1X) at 37°C in 5% CO_2_.

### Animals

Animal experiments were approved by the Danish Animal Experiments Inspectorate (Copenhagen, Denmark) and performed according to the Danish Act on Animal Experiments (LBK No. 474 of 15/05/2014) and in compliance with the *Guide for the Care and Use of Laboratory Animals: Eighth Edition* (National Academies Press, Washington, DC, USA; 2011). Sixty‐five 4‐ to 5‐week‐old male BKAL mice (Envigo, Venray, Netherlands) were housed in groups of four or five in individually ventilated GM500+ cages (524 cm^2^), with standard diet (Altromin 1314; Brogaarden, Gentofte, Hovedstaden, Denmark) and tap water *ad libitum*, in a temperature‐controlled room (21°C to 24°C) on a 12‐hour light/dark cycle. Cages contained Tapvei 2 HV bedding (Brogaarden) and environmental enrichment: S‐brick (Tapvei, Paekna, Estonia), red translucent shelter, paper ropes, and corn hidden in the bedding. Mice were randomized into sham or myeloma‐bearing groups according to weight or burrowing capacity. General health, including body weight, coat condition, and abnormal behaviors, was regularly assessed.

### Animal models

The systemic model of MM was generated by i.v. injection of 5TGM1‐GFP cells (2 × 10^6^/100 μL; *n* = 24) or vehicle (saline; *n* = 25) into the tail veins of 5‐ to 6‐week‐old awake BKAL mice; in the localized model, 5TGM1‐GFP cells (5 × 10^4^/10 μL; *n* = 29) or vehicle (saline; *n* = 16) were inoculated in the intramedullary femoral cavity, as previously described in models of cancer‐induced bone pain.^24^ Briefly, 5‐ or 6‐week‐old mice were rapidly anesthetized with 4% isoflurane (Nomeco, Copenhagen, Denmark), and thereafter administered an i.p. cocktail of ketamine/xylazine [85.5 mg/kg ketamine from Ketaminol vet (MSD Animal Health, AN Boxmeer, The Netherlands) and 12.5 mg/kg xylazine from Nerfasin vet (Virbac, Kolding, Denmark)]. Full anesthesia was confirmed by loss of pedal reflex; eye ointment (Ophta A/S, Actavis Group, Gentofte, Denmark) was applied and the mouse was placed on a heating pad on the dorsal side; the right anterior knee was shaved and disinfected with 70% ethanol. An incision (<10 mm) was made on the superficial skin covering the patella, the retinaculum tendon was loosened and the patella displaced, exposing the distal femoral epiphysis. A 30G needle was used to drill a hole into the epiphysis, and then an insulin needle (0.3 mL; BD Rowa Technologies [BD], Lyngby, Denmark) was inserted. Vehicle (10 μL PBS) or 5 × 10^4^ 5TGM1‐GFP cells/10 μL PBS were injected into the femoral intramedullary cavity. The hole was closed with Ethicon bone wax (Mediq Danmark A/S, Brøndy, Denmark), and the patella was carefully pushed into place. The wound was irrigated with saline and closed with two surgical clips (7.50 × 1.75 mm; Agnthos, Lidingö, Sweden). Mice received s.c. administration of 500 μL saline and 0.03 mg/kg buprenorphine (Temgesic; Indivior UK Limited, Slough, UK), and xylocaine gel (2% w/v, Nomeco) was applied on the surgical site. Mice were randomized into sham or myeloma‐bearing groups according to weight or burrowing performance.

### Behavioral testing

All behavioral tests were performed by the same researchers, who were blinded to the experimental groups/conditions, between 8:00 a.m. and 2:00 p.m.

### Stimulus‐evoked behavioral tests

#### *von Frey test*

Mechanical hypersensitivity was assessed with von Frey filaments following the Dixon up‐and‐down method^25^ to calculate the 50% paw withdrawal threshold as defined by Chaplan and colleagues.^26^, ^27^ Briefly, mice were acclimatized to individual chambers (90  × 90 × 120 mm), placed on an elevated wire grid for 30 to 60 min, and von Frey monofilaments (North Coast Touch Test; North Coast Medical, Inc., Morgan Hill, CA, USA) applied perpendicularly on the plantar surface of the right hind paw for 3 s. Every animal was tested in a range between 0.04 and 4.00*g*, with a starting point of 0.60*g*; increasing forces were used if a negative response was recorded, whereas decreasing forces were used following a positive response. Positive responses were defined as clear withdrawal of the limb following exposure to a filament. Animals were subjected to two training (including chamber acclimation and random poking) and two baseline sessions.

#### *Hot plate test*

Thermal hyperalgesia was evaluated on a Hot Plate Analgesia Meter (Harvard Apparatus, Holliston, MA, USA) set at 52.5 ± 0.1°C, by quantifying the time lapse between animal exposure to the apparatus and a positive response. Positive responses were defined as jumping and licking or flinching of either hind paw (systemic model) or the right hind paw (localized model); as previously described,^28^ a cut‐off of 40 s was established to avoid tissue damage.

### Non‐stimulus‐evoked behavioral tests

#### *Burrowing*

Burrowing capacity was assessed as previously described,^29^ by individually exposing the mice to burrowing tubes (200 mm length × 72 mm diameter, raised 30 mm from the ground in the frontal part) filled with 500 g sand (0–3 mm diameter, ScanSand, Herlev, Denmark) placed in standard plastic cages without bedding (125 × 66 × 185 mm). Initially, mice were habituated to the apparatus by placing them in pairs in a box with an empty burrowing tube. On the second and third training days, mice were again placed in pairs with the same burrowing tube, filled with 500‐g sand. Two baseline testing sessions were conducted in the following days by placing the mice individually within a burrowing tube filled with 500‐g sand and the amount of sand left in the tube was weighed after each session. Thereafter, the same setup was used on testing days. Burrowing performance was calculated as 500 g minus the amount of sand left in the tube (g) at the end of the burrowing session; all burrowing sessions lasted 2 hours.

#### *Locomotor activity*

Locomotor activity of freely walking mice was individually measured in standard plastic cages without bedding (125 × 266 × 185 mm) with the tracking software Ethovision XT 8.5 (Noldus Information Technology, Wageningen, The Netherlands); initially, mice were acclimatized for 15 min, and the speed and distance walked for the following 15 min were calculated.

#### *Limb use*

Limb use was evaluated as previously described^30^ by observing the mice freely walking in standard plastic cages (125 × 266 × 185 mm) without bedding for 3 min after 10‐min acclimation. The scoring was: 4 = normal gait, 3 = insignificant limping, 2 = significant limping accompanied by a shift in body distribution towards the healthy limb, 1 = significant limping and partial paraplegia, 0 = paraplegia (defined as humane endpoint).

### Morphine validation experiment

On postsurgical day 26, the limb use score of all animals was assessed. Thereafter, i.p. saline (100 μL) was administered to all mice and limb use was assessed after 30, 60, and 120 min. Mice were returned to their cages for 4 hours, and their limb use was assessed again. Then, all animals received 10 mg/kg^−1^ morphine^30^ (morphine hydrochloride; Skanderborg Produktionsapotek, Copenhagen, Denmark), dissolved in i.p. saline (100 μL) and their gait was evaluated after 30, 60, and 120 min. To avoid a ceiling effect, myeloma‐bearing animals with a limb use score of 4 on day 26 were excluded, as a score of 4 in the limb use test indicates the absence of a pain‐like phenotype, and therefore the lack of a therapeutic window (*n* = 3); similarly, myeloma‐bearing mice with a limb use score ≤1, which could be indicative of motor impairment caused by paraplegia development, were also excluded (*n* = 2). Experimenters were completely blinded to the study design, and the exclusion parameters were carried out by a different researcher than the one performing all behavioral tests.

### Bisphosphonate experiment

Mice were treated biweekly with vehicle (sham *n* = 4 and MM *n* = 8) or zoledronic acid (125 μg/kg^−1^, Procter and Gamble Pharmaceuticals, Mason, OH, USA; *n* = 7), administered s.c. in a volume of 100 μL, as previously described.^31^ Treatment started 3 days after tumor inoculation and lasted for the duration of the experiment. Animals were randomized according to weight into the different treatment groups. All dosing and behavioral testing was conducted by an experimenter blinded to the experimental groups and treatments.

### Euthanasia and tissue analyses

Mice were euthanized by cervical dislocation, the spleens weighed, and tibias and femurs excised. The joints were cut, and the bone marrows extruded with 500‐μL PBS through a 27G needle. Blood cells were lysed with the Human Erythrocyte Lysing Kit (R&D Systems, Minneapolis, MN, USA) following the manufacturer's recommendations; the resulting cell suspension was imaged in a ZOE Fluorescent Cell Imager (Bio‐Rad Laboratories, Hercules, CA, USA). Cells were automatically quantified with ImageJ software (NIH, Bethesda, MD, USA; https://imagej.nih.gov/ij/) and the percentage of GFP‐positive cells calculated as the average of three pictures.

Alternatively, mice were transcardially perfused with ice‐cold PBS and 25 to 30 mL of 4% paraformaldehyde (Merck, Søborg, Denmark). Hind limb bones were postfixated in 4% paraformaldehyde at 4°C for 24 hours and stored in 0.1M PBS or 70% ethanol at 4°C.

### Determination of serum levels of paraprotein by ELISA

Serum samples were obtained by cardiac puncture at euthanasia. The serum IgG_2b_ paraprotein content was assessed in an ELISA kit (Cat# E99‐109; Bethyl Laboratories, Inc., Mountain View, CA, USA) following manufacturer's recommendations.

### Micro‐computed tomography (μCT) analyses

μCT scanning and lesion analyses were performed using a SkyScan 1272 Desktop X‐Ray Tomographer (SkyScan, Aartselaar, Belgium) as previously described.^31^ Bone disease was assessed by μCT measuring of osteolytic lesion area (%), trabecular bone volume (% BV/TV), trabecular thickness (Tb. Th), and trabecular number (Tb. N) using a SkyScan 1272 Desktop X‐Ray Tomographer with a medium‐sized 2000 × 1048 pixel camera and an X‐ray source powered by 50 kV and 200 μA electric current. Low‐energy radiations were filtered with a 0.5‐mm aluminum filter. The proximal end of the tibia or distal femur was scanned at 4.3‐μm pixels for every 0.7‐degree rotation of 180 degrees. The scanned images were reconstructed using NRecon software (version 1.6.1.1; SkyScan) within a dynamic range of 0 to 0.14 and a ring artifact reduction factor of 1%. The reconstructed images were analyzed using CTAn (version 1.9.1.1 Bruker, Belgium). Analysis were done on the cross‐sectional images of the tibias or femurs at a distance of 1.2 mm (offset) from the distal break in the growth plate (reference point), on a fixed 1‐mm region. The trabecular bone was carefully traced on all cross‐sectional images of the tibia, not to include cortical bone for the entire ROI. Analyses were done in batches using BatMan (Batch Manager Bruker, Belgium) software; for trabecular analysis, the binarized images were thresholded between 90 to 255.

### Immunohistochemistry and laser confocal microscopy

Femurs were gently decalcified in 10% EDTA for approximately 2 weeks at 4°C; decalcification was monitored radiographically to ensure minimum EDTA exposure and maximum immunohistochemical antigenicity. Next, femurs were cryoprotected in 30% sucrose for 48 hours and serially sectioned in a cryostat along the longitudinal axis at a thickness of 20 μm. Sections were blocked (3% normal donkey serum, 0.3% triton X‐100) for 1 hour and incubated with a cocktail of anti‐GFP antibody (polyclonal chicken anti‐GFP, 1:2000; Cat #A10262; Thermo Fisher Scientific, Rockford, IL, USA) and antibodies against calcitonin‐gene‐related peptide (CGRP; polyclonal rabbit anti‐rat CGRP, conjugated to keyhole limpet hemocyanin^32^, ^33^, ^34^ 1:5000; #C8198; Sigma‐Aldrich, St. Louis, MO, USA), growth‐associated protein‐43 (GAP43; rabbit anti‐GAP43; #AB5220; Millipore, Billerica, MA, USA), or tyrosine hydroxylase (TH; polyclonal rabbit anti‐rat TH, 1:1000; #AB152; Chemicon, Billerica, MA, USA). Preparations were washed three times in PBS, incubated with a cocktail of Cy2/Cy3 secondary antibodies (1:400/1:600; Jackson ImmunoResearch, West Grove, PA, USA) for 3 hours, washed with PBS three times, counterstained with 4′, 6‐diamidino‐2‐phenyl‐indole, dihydrochloride (DAPI; 1:30,000; Molecular Probes, Eugene, OR, USA) for 5 min, and washed with PBS.

Tissue was dehydrated through an alcohol gradient (70%, 80%, 90%, and 100%), cleared in xylene, coverslipped with di‐n‐butylphthalate‐polystyrene‐xylene (Sigma‐Aldrich) and allowed to dry at room temperature for 12 hours. Sections were imaged in a scanning confocal laser microscope (Carl Zeiss, model LSM800; Jena, Germany). Sequential acquisition mode was used to reduce bleed‐through from fluorophores.

### Statistical analyses

Data were analyzed in GraphPad Prism 7.0 (GraphPad Inc., La Jolla, CA, USA) or SAS 9.4 (SAS Institute, Inc., Cary, NC, USA). The experimental unit was a single animal. Sample size was determined empirically from experience with other models of cancer‐induced pain. Parametric behavioral data were analyzed by two‐way repeated measurements ANOVA with Bonferroni correction; limb use data were analyzed by Friedman's two‐way test and Wilcoxon's two‐sample test for single timepoints or by transforming the data to area under the curve followed by one‐way ANOVA and then Holm‐Sidak correction for multiple comparisons. Morphine treatment was analyzed by Friedman's two‐way test and the difference between morphine‐treated and saline‐treated animals with Wilcoxon's two‐sample test for each timepoint. Last measurements were carried forward, except in the bisphosphonate experiment. Nonbehavioral data were analyzed by single or multiple two‐tailed, unpaired Student's *t* tests. All data (except survival percentage) are presented as mean ± SEM. Statistical significance was set at 0.05.

### Data‐sharing statement

All original protocols used and data reported in this publication are available upon request. For original data, please contact: marta.castillo@sund.ku.dk.

## Results

### Systemic inoculation of myeloma cells does not induce pain‐like behaviors

**Figure 1:**
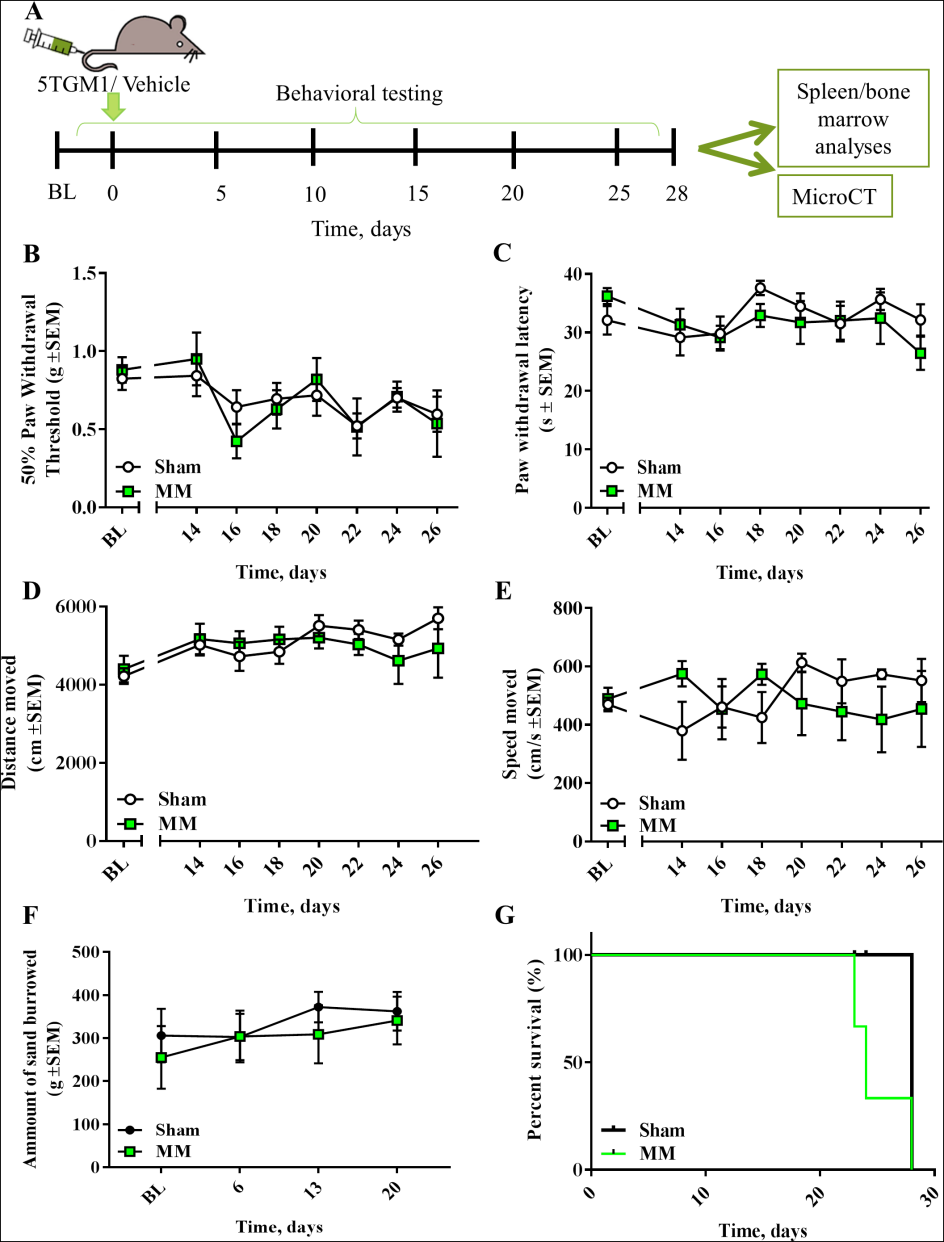
The systemic 5TGM1 model does not present pain‐like behaviors (*A*) 5TGM1 myeloma cells or vehicle was administered to BKAL mice through tail vein injection; thereafter, behavioral tests were conducted for 4 weeks. Upon euthanasia, hind limb bones were used to assess the presence of myeloma cells or of osteolytic lesions. (*B*) Paw withdrawal threshold, as measured in the von Frey test, remained unchanged between myeloma‐bearing (*n* = 5) and sham (*n* = 8) mice over time. (*C*) Paw withdrawal latency, measured in the hot plate test, of myeloma‐bearing (*n* = 5) and sham (*n* = 8) mice was not altered over time. (*D*,*E*) similar distance (*D*) and speed (*E*) moved in the locomotor test by myeloma‐bearing (*n* = 5) and sham (*n* = 8) mice over time. (*F*) Amount of sand burrowed by myeloma‐bearing (*n* = 8) and sham (*n* = 9) mice over time. (*G*) Survival plot of myeloma‐bearing (*n* = 5) and sham (*n* = 8) mice, depicted as percentage of survival.

To assess pain‐related behaviors in a widely used syngeneic model of MM, we inoculated 5TGM1‐GFP myeloma cells or vehicle into the tail vein of BKAL mice (Fig. 1 *A*) and assessed them in stimulus‐ and non‐stimulus‐evoked behavioral tests. von Frey and hot plate tests (stimulus‐evoked tests; Fig. 1 *B*,*C*) showed no differences in mechanical allodynia or thermal hyperalgesia; likewise, no differences in burrowing capacity or locomotor activity (non‐stimulus‐evoked tests; Fig. 1 *D*–*F*) were observed in this mouse model, indicating that myeloma‐bearing mice did not present quantifiable pain‐related behaviors.

### Systemic inoculation of myeloma cells induces the disease phenotype, but not osteolytic lesions

**Figure 2:**
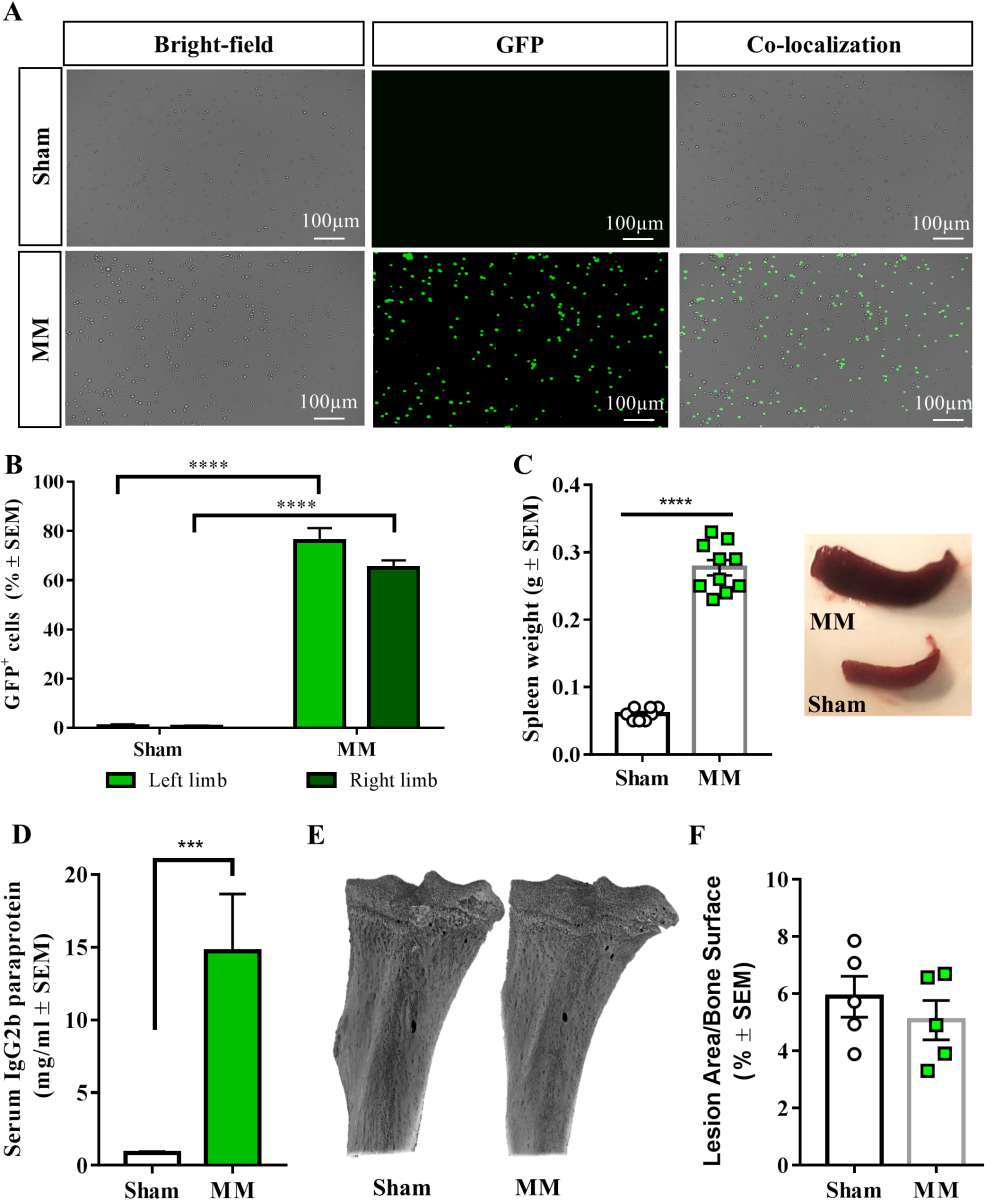
Systemic inoculation of 5TGM1 cells induces myeloma, but not osteolytic lesions. (*A*) Microscopic imaging of bone marrow flushes demonstrated the presence of myeloma cells (green fluorescent protein‐ (GFP‐) positive) in the long bones of 5TGM1‐inoculated, but not sham, mice. Images were captured in a ZOE Fluorescence Cell Imager (objective 20×; bright field channel LED intensity 40 and exposure time 300 ms; green channel LED intensity 50 and exposure time 500 ms); image contrast was enhanced for better visualization of GFP‐positive cells in ImageJ. Scale bars represent 100 μm. (*B*) Quantification of GFP‐positive cells in the bone marrow of right and left hind limbs of myeloma‐bearing and sham mice (*n* = 8–10; ****p < 0.0001). (*C*) Myeloma‐bearing mice presented splenomegaly compared with sham mice (*n* = 8–10; ****p < 0.0001). (*D*) The concentration of serum paraprotein at euthanasia was significantly higher in systemically inoculated myeloma‐bearing mice than sham (*n* = 5–7; ***p < 0.001). (*E*) Three‐dimensional μCT reconstructions of the tibia of myeloma‐bearing and sham mice. (*F*) Myeloma‐bearing mice did not present osteolytic lesions compared with sham. MM = multiple myeloma.

The MM phenotype was confirmed by development of paraplegia by myeloma‐bearing mice, which was defined as a humane endpoint, approximately 3 to 4 weeks after cell inoculation (Fig. 1 *G*). To further demonstrate disease development, spleen weight and the presence of myeloma cells in the bone marrows were assessed. As expected, microscopic analyses demonstrated extensive bone marrow invasion by 5TGM1‐GFP myeloma cells (Fig. 2 *A*,*B*). Moreover, myeloma‐bearing mice presented splenomegaly, with significantly heavier spleens than their matched controls (Fig. 2 *C*), as well as increased serum paraprotein (Fig. 2 *D*).

Although systemic administration of 5TGM1‐GFP cells induced the myeloma‐like phenotype, μCT scanning of the long bones, a common site of myeloma development in patients and mice,^35^, ^36^ showed that myeloma‐bearing tibias did not display significant osteolytic lesions (Fig. 2 *E*,*F*). As the development of bone pain has often been associated with the presence of osteolytic lesions,^37^, ^38^ these results are consistent with the behavioral data showing lack of quantifiable pain‐related behaviors in this model.

### Intrafemoral inoculation of myeloma cells induces osteolytic lesions

**Figure 3:**
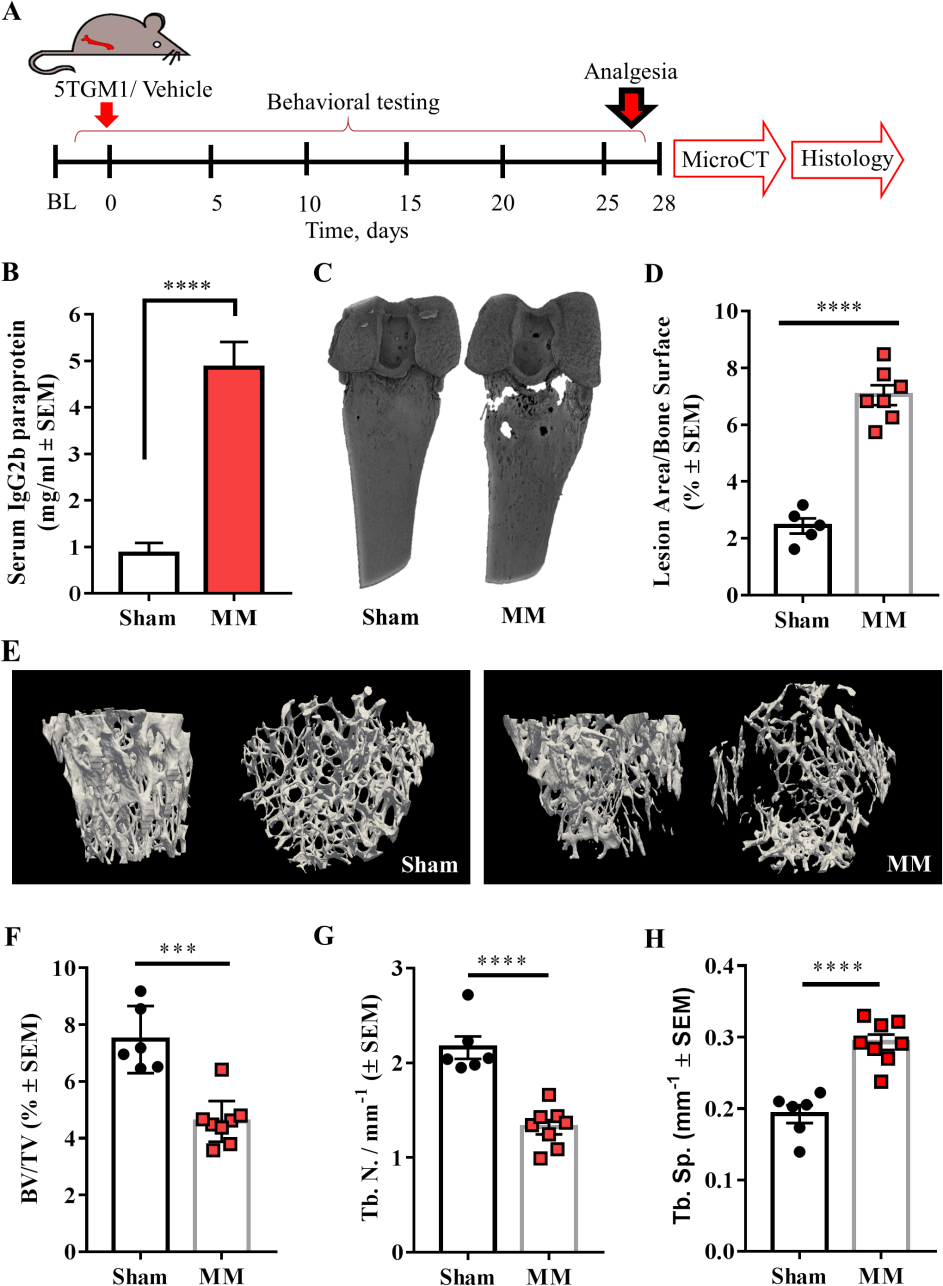
Intrafemoral inoculation of 5GTM1 cells induces osteolytic lesions. (*A*) 5TGM1 myeloma cells or vehicle were inoculated in the medullary femoral cavity of BKAL mice; thereafter, behavioral tests were conducted for the following 4 weeks. Upon euthanasia, mice were perfused and their femurs analyzed by μCT and immunohistochemistry. (*B*) The concentration of serum paraprotein at euthanasia was significantly higher in intrafemorally inoculated myeloma‐bearing mice than in sham mice (*n* = 6–7; ****p < 0.0001). (*C*) Three‐dimensional μCT reconstructions of the femur of myeloma‐bearing and sham mice. Note the presence of osteolytic lesions in myeloma‐inoculated femurs. (*D*) Lesion area analyses of myeloma‐bearing (*n* = 7) and sham (*n* = 5) femurs. (*E*) Three‐dimensional reconstruction of the trabecular bone of sham and myeloma‐bearing bones. (*F*) Myeloma bearing femurs (*n* = 8) presented decreased bone volume/total volume than sham bones (*n* = 6; ***p < 0.001). (*G*) The trabecular number of myeloma‐bearing femurs (*n* = 8) was significantly lower than that of sham femurs (*n* = 6; ****p < 0.0001). (H) Myeloma‐bearing femurs (*n* = 8) showed significantly higher trabecular separation than sham femurs (*n* = 6; ****p < 0.0001). MM = multiple myeloma.

As the systemic myeloma model did not display pain‐like behaviors or extensive osteolytic lesions, we investigated a localized model of MM by administering 5TGM1‐GFP cells into the intramedullary femoral cavity of BKAL mice (Fig. 3 *A*). Disease phenotype was confirmed by significantly increased levels of systemic serum paraprotein in myeloma‐bearing mice, compared with sham (Fig. 3 *B*). In these animals, μCT analyses of the femurs showed a threefold increase in the osteolytic lesion area (Fig. 3 *C*,*D*), as well as a significant decrease in % BV/TV, Tb. N, and an increase in trabecular separation, compared with control mice (Fig. 3 *E*–*H*). Importantly, osteolytic lesion development did not occur at the injection site (ie, at the site where the needle drilled into the bone); these results demonstrate that intrafemoral inoculation of 5TGM1‐GFP induces extensive myeloma bone disease in the localized 5TGM1 model.

### Intrafemoral inoculation of myeloma cells induces pain‐like behaviors

**Figure 4:**
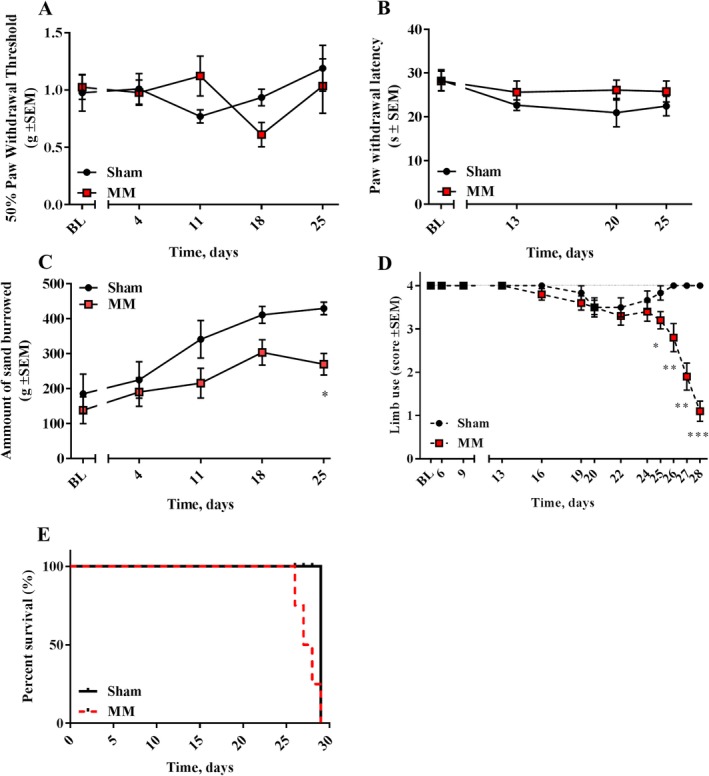
The localized 5TGM1 model presents pain‐like behaviors (*A*) Paw withdrawal threshold, as measured in the von Frey test, remained unchanged between myeloma‐bearing (*n* = 10) and sham (*n* = 6) mice over time. (*B*) Paw withdrawal latency, measured in the hot plate test, of myeloma‐bearing (*n* = 10) and sham (*n* = 6) mice was not altered over time. (*C*) Myeloma‐bearing mice (*n* = 10) burrowed a significantly lower amount of sand compared with sham (*n* = 6), 25 days after surgery (*p < 0.05). (*D*) The limb use score of myeloma‐bearing mice (*n* = 10) decreased over time, compared with sham (*n* = 6; *p < 0.05; **p < 0.01; ***p < 0.001) (*E*) Survival plot of myeloma‐bearing (*n* = 10) and sham (*n* = 6) mice, depicted as percentage of survival. MM = multiple myeloma.

To evaluate whether myeloma‐induced bone disease in the localized model would cause the development of pain‐related behaviors, mice were exposed to stimulus‐evoked and non‐stimulus‐evoked behavioral tests. Similar to the systemic model, mechanical allodynia and thermal hyperalgesia were unchanged in myeloma‐bearing mice (Fig. 4 *A*,*B*). However, non‐stimulus‐evoked behavioral tests showed a reduction in burrowing capacity, as well as a decrease in limb use score in myeloma‐bearing mice, compared with their sham matched controls (Fig. 4 *C*,*D*).

**Figure 5:**
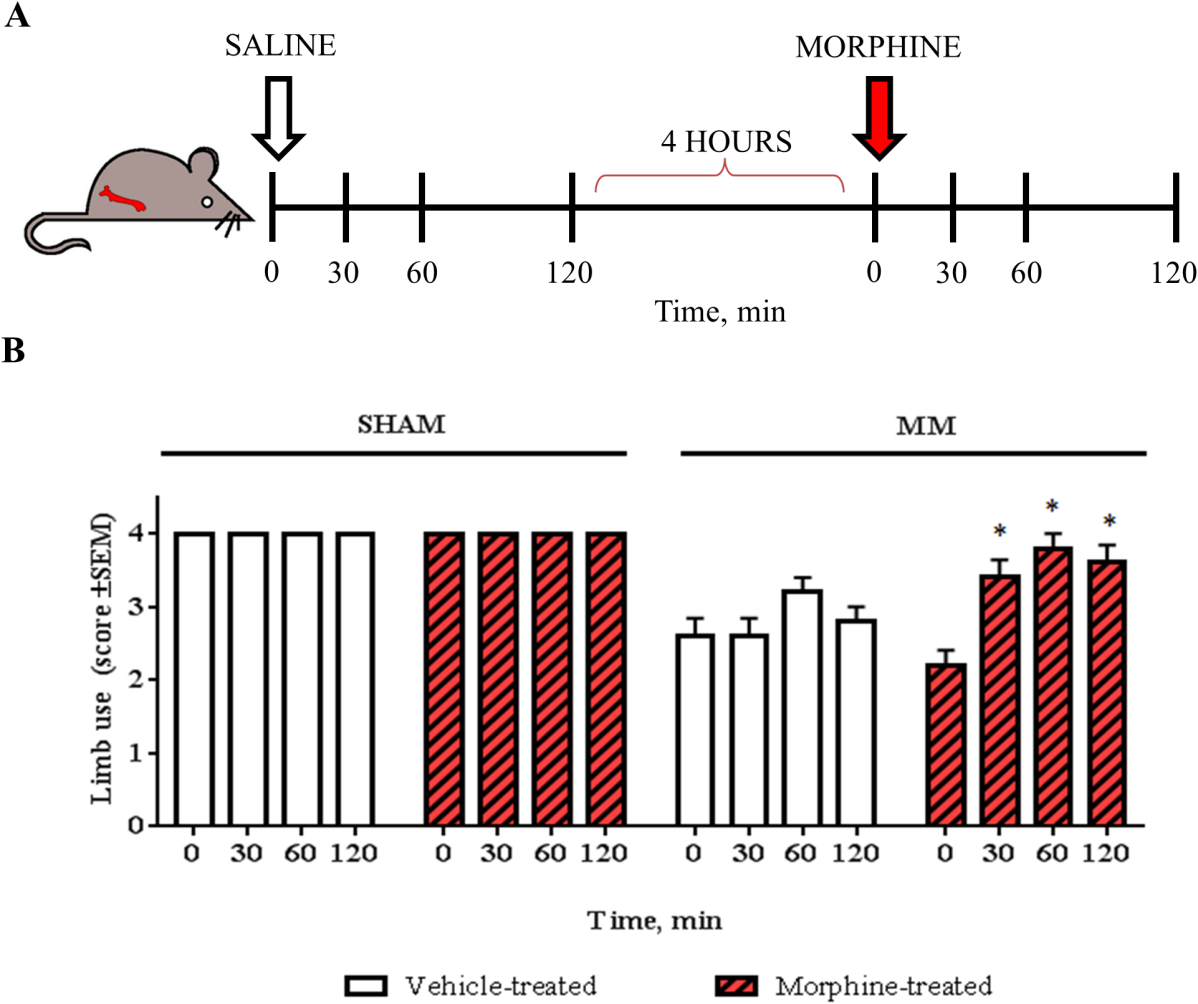
Systemic morphine reverses pain‐like behaviors in the localized 5TGM1 model. (*A*) Myeloma‐bearing and sham mice were assessed in the limb use test, 26 days after surgery. Thereafter, all animals received i.p. saline and their limb use score was assessed in the following 30, 60, and 120 min. After 4 hours from the last assessment, the limb use was again scored and all animals received i.p. morphine (10 mg/kg). The limb use test was then conducted 30, 60, and 120 min after morphine administration. (*B*) Myeloma‐bearing mice showed a significant increase in limb use scores 30, 60, and 120 min after morphine administration, compared with vehicle (*n* = 5–6; *p < 0.05, **p < 0.01). MM = multiple myeloma.

Myeloma‐bearing mice developed paraplegia approximately 3 to 4 weeks after cell inoculation, further confirming the disease phenotype (Fig. 4 *E*). To demonstrate that the behavioral outcomes were caused by myeloma‐induced bone pain and not by motor impairment as a consequence of disease development, we evaluated the analgesic effect of morphine in this model 26 days after cell inoculation. The effect of systemic morphine, at a dose comparable to that used for the treatment of cancer‐induced bone pain in murine models (10 mg/kg^−1^),^30^ was examined in the limb use test (Fig. 5A). Morphine treatment improved the limb use scores of myeloma‐bearing animals, demonstrating that intrafemoral inoculation of myeloma cells induces a pain‐like phenotype, which is reversible by a clinically relevant opioid (Fig. 5 *B*).

### Bisphosphonates prevent myeloma bone disease and attenuate pain‐like behaviors

**Figure 6:**
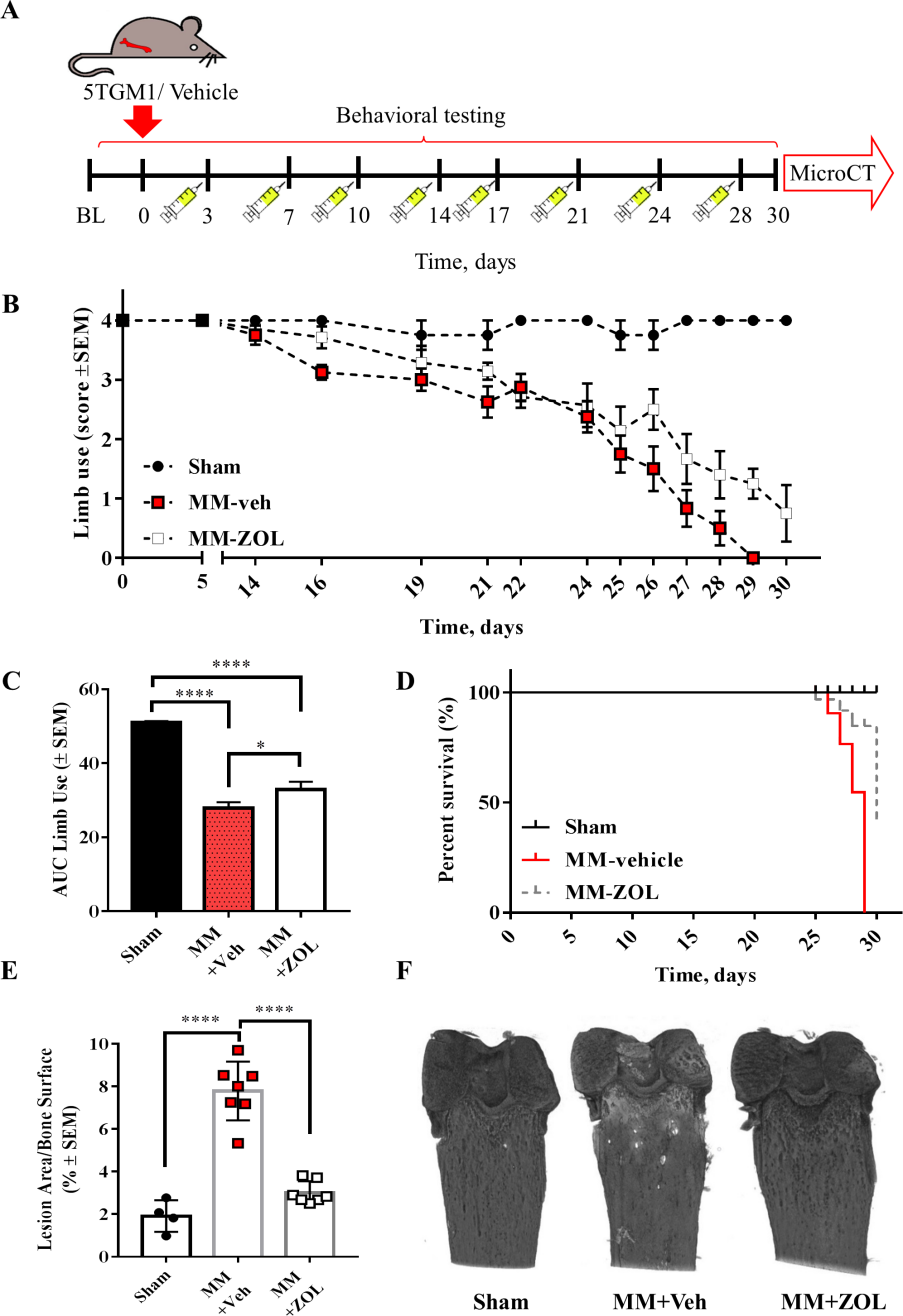
Bisphosphonates prevent myeloma bone disease and have an analgesic effect on the localized 5TGM1 model. (*A*) 5TGM1 myeloma cells or vehicle were inoculated in the medullary femoral cavity of BKAL mice; thereafter, behavioral tests were conducted for the following 30 days. Animals were dosed with vehicle or zoledronic acid (125 μg/kg^−1^) on postsurgical days 3, 7, 10, 14, 17, 21, 24, and 28. (*B*,*C*) myeloma‐bearing mice (*n* = 7) treated biweekly with zoledronic acid (125 μg kg^−1^) showed an amelioration of pain‐like behavior, compared with vehicle‐treated, myeloma‐bearing mice (*n* = 8). Both groups showed significant pain‐like behavior compared with sham‐operated mice (*n* = 4; *p < 0.05, ****p < 0.0001). (*D*) Bisphosphonate‐treated, myeloma‐bearing mice showed increased survival compared with vehicle‐treated, myeloma‐bearing mice. (*E*) Myeloma‐bearing, bisphosphonate‐treated femurs (*n* = 7), like sham (*n* = 4), presented significantly less osteolytic lesions that myeloma‐bearing, vehicle‐treated femurs (*n* = 7; ****p < 0.0001). (*F*) Three‐dimensional μCT reconstructions of the femur of sham and myeloma‐bearing, vehicle‐ or bisphosphonate‐treated femurs. MM + veh = Myeloma‐bearing, vehicle‐treated MM + ZOL = myeloma‐bearing, zoledronic acid‐treated.

To examine whether bone disease was the main driver of the pain‐like phenotype, myeloma‐bearing mice were treated twice a week with vehicle or zoledronic acid (125 μg/kg^−1^; Fig. 6 *A*) as previously described.^39^ Biweekly bisphosphonate treatment caused a significant attenuation of pain‐like behavior over time as assessed by the limb use test (Fig. 6 *B*,*C*), as well as an increase in survival rate (Fig 6 *D*). Analysis of bone lesions showed that biweekly bisphosphonate treatment effectively protects the myeloma‐bearing bones from developing osteolytic lesions (Fig. 6 *E*,*F*).

### Intrafemoral inoculation of myeloma cells causes denervation

**Figure 7:**
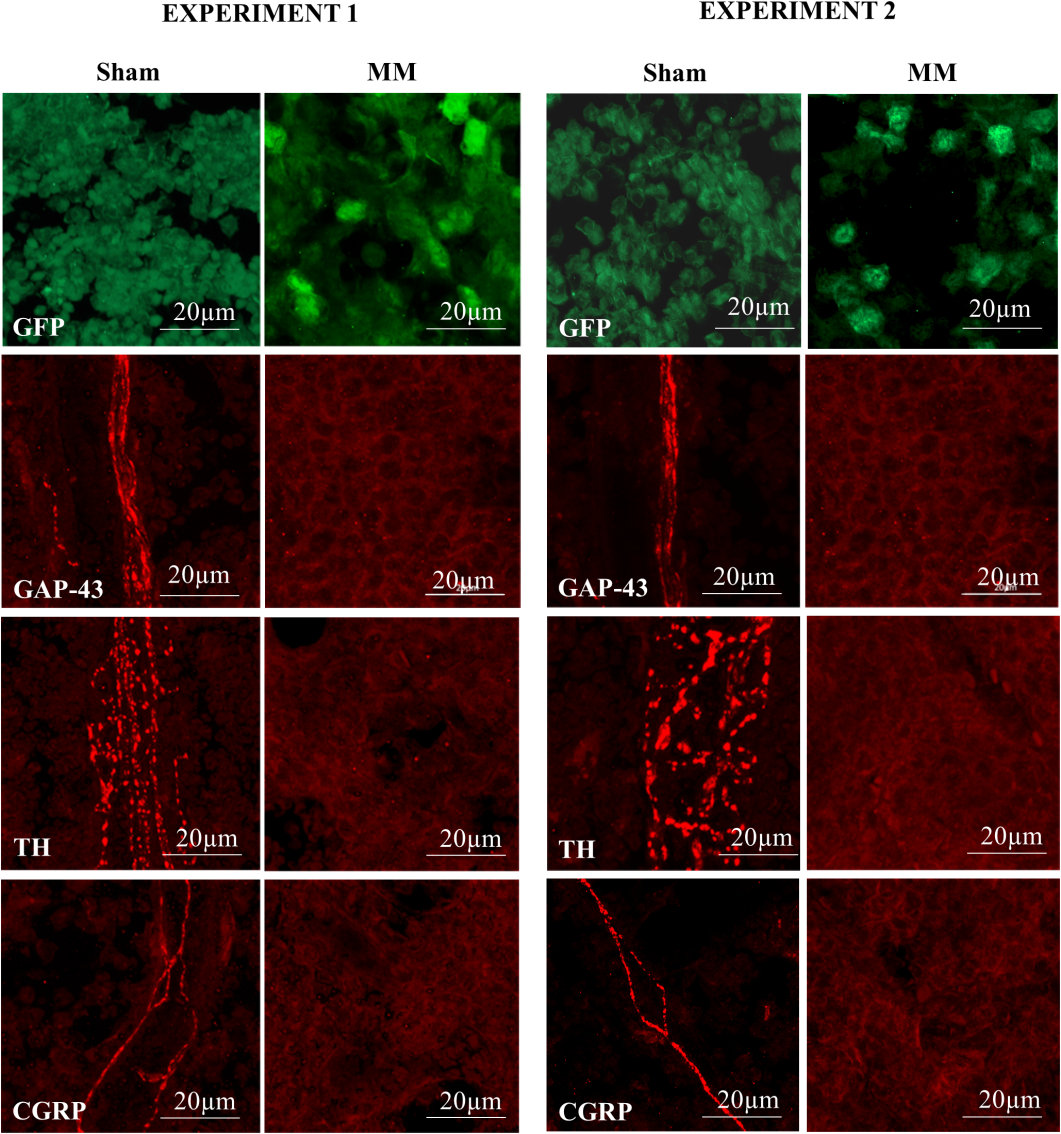
The localized 5TGM1 presents denervation of the bone marrow. Representative pictures of histological sections of myeloma‐bearing and sham femurs, stained for GFP (marker of myeloma cells, 5TGM1‐GFP), GAP‐43 (marked or nerve growth), TH (marker of sympathetic neurons), and CGRP (marker of sensory neurons). In two independent experiments, myeloma‐bearing femurs showed extensive denervation of the bone marrow compared with sham. Images were captured in a Carl Zeiss scanning confocal laser microscope (model LSM800) with a Plan‐Apochromat 63×/1.40 oil differential interference contrast (DIC) (working distance = 0.19 mm) objective and a numerical aperture of 1.4, at 23°C. Images were taken with the X‐stack function at an interval of 1.00 μm with a Carl Zeiss model Axio Cam ICc5 camera, and the software Zen 2.3 blue edition system (Zeiss). The used fluorochromes were Cy3‐conjugated affiniPure donkey anti‐rabbit IgG and Cy2‐conjugated affinPure donkey anti‐chicken IgG. Scale bars represent 20 μm.

To further ratify the disease phenotype, the presence of 5TGM1‐GFP cells in the bone marrow of myeloma‐inoculated mice was evaluated by immunohistochemistry in decalcified bone sections. GFP‐positive cells were found extensively infiltrating the bone marrow of myeloma‐bearing femurs, demonstrating tumor cell growth in this model (Fig. 7).

Moreover, to investigate whether tumor growth affected the number and distribution of nerve fibers, broadly characterized immunohistochemical markers for neuronal growth (GAP‐43), sensory (TH), and sympathetic (CGRP) neurons were assessed by immunohistochemistry and laser confocal microscopy. Although all three neuronal markers were detectable in the bone marrow of femurs from sham mice, myeloma‐bearing femurs did not display detectable staining, indicating complete denervation of the bone marrow of these mouse bones. These results were consistent and reproducible between experiments, and suggest that myeloma growth induces bone marrow denervation in this model (Fig. 7).

## Discussion

Bone pain is a serious and debilitating symptom for MM patients^12^; however, it remains understudied and its causes are unknown. Current therapies targeting cancer pain are based on pain intensity^40^ rather than its underlying mechanisms, leading to analgesic treatments with low efficacy and unwanted side‐effects. Superior drugs targeting the disease‐specific mechanisms of bone pain are crucial to improve the quality of life of MM patients. Here, we present an immunocompetent mouse model of myeloma‐induced bone pain and show that morphine reverses its pain‐like phenotype, suggesting its adequacy for pharmacological testing of novel therapies. Additionally, we propose that myeloma‐induced bone pain is driven by the combinatorial effect of osteolytic lesion development and bone marrow denervation.

Although many preclinical models of MM are available,^36^ currently only one study by Hiasa and colleagues^(14)^ has addressed pain‐related behaviors, but their results are mainly based on a xenograft myeloma model, which uses immunocompromised mice. However, mounting evidence has shown that the immune system plays a pivotal role in the transition from acute to chronic pain,^41^, ^42^ suggesting that the validity of xenograft models is compromised. In contrast, we investigated pain‐related behaviors in the immunocompetent 5TGM1‐GFP model, in which syngeneic myeloma cells are systemically administered in the tail vein.^19^ Although the disease phenotype in this model was confirmed by increased serum paraprotein, splenomegaly, tumor development, and paralysis, pain‐related behaviors were not apparent in these mice. Additionally, significant bone disease was not detected, in agreement with previous reports,^43^ therefore highlighting that osteolysis is highly variable in this systemic model.

We investigated a localized model of MM in which intrafemoral inoculation of 5TGM1‐GFP cells in BKAL mice induces quantifiable pain‐related behavior and osteolytic lesions. Mechanical and thermal thresholds were unchanged in myeloma‐bearing mice; however, it has previously been shown that these tests might not be appropriate measures of deep tissue pain such as bone pain.^44^ Instead, more appropriate approaches are tests that measure etiologically relevant behaviors indicative of well‐being and pain‐related behavior in rodents,^45^, ^46^ such as burrowing and limb use. Using these tests, we demonstrated the progressive development of a bone pain‐like phenotype in myeloma‐bearing mice. Moreover, this phenotype could be reversed by a conventional opioid commonly used in the clinic,^40^ further demonstrating that these readouts are measures of pain‐related behavior rather than motor impairment. Additionally, reversal of the pain‐like phenotype by morphine confirms the validity of this model for testing novel analgesic drugs with translational potential.

Intrafemoral inoculation of 5TGM1 myeloma cells led to the stable development of osteolytic lesions, similar to those found in the clinic.^47^ Moreover, intrafemoral inoculation of myeloma cells in BKAL mice led to the development of paraplegia approximately 4 weeks after cell inoculation, indicating that the myeloma cells may have migrated to the vertebral bodies, leading to spinal compression and consequent paraplegia, a well‐described feature of the disease in patients^5^ that supports the translational validity of this model. Although these mice showed a significant increase in serum paraprotein, the paraprotein levels were lower than those detected in the serum of systemically inoculated mice. This is likely explained by the confined tumor environment following the intrafemoral inoculation of myeloma cells in this model, compared with the systemic one.

The effect of a common bisphosphonate, zoledronic acid, was evaluated in the localized 5GTM1 model. Bisphosphonate treatment is commonly used in the clinic to improve bone quality and decrease skeletal‐related events in myeloma patients, and systematic literature reviews have shown that it ameliorates pain^48^, ^49^ However, the quality of the pain data has recently been questioned because of the different read‐outs used in different trials and the lack of clinically significant analgesic effect in double‐blinded clinical trials.^50^ In the localized 5TGM1 model, systemic treatment with zoledronic acid protected the myeloma‐bearing bones from developing osteolytic lesions. Bisphosphonate treatment also led to increased survival and partly ameliorated the pain‐like behavior over time, suggesting that osteolytic bone lesions may play a role in pain development, but not be entirely responsible for it. The fact that the localized MM model displayed pain‐like behaviors whereas the systemic model did not, could thus be partially explained by the lack of osteolytic lesion development in the latter. However, it should be considered that the systemic model may display nociception that could not be detected with the battery of behavioral tests used in this study.

Interestingly, complete denervation of the bone marrow of myeloma‐bearing femurs was consistently observed across experiments demonstrating, for the first time, that myeloma tumor growth induces damage of the sensory and sympathetic nerves, possibly contributing to pain development through injury‐induced neuronal sensitization. Our results are in agreement with previously published reports of bone marrow denervation in a mouse model of osteosarcoma^51^; here, sarcoma‐inoculated mice also showed a decrease in limb use scores that were accompanied by multiple neurochemical changes in the cell bodies of the sensory neurons, potentially as a consequence of tumor‐induced bone marrow denervation. Interestingly, the only available report describing the sensory innervation of myeloma‐bearing femurs shows neuronal sprouting^14^ parallel to disease development, which has also been described in other models of cancer‐induced bone pain.^52^, ^53^ Although it cannot be ruled out that myeloma‐inoculated mice also presented nerve sprouting prior to spinal compression and paraplegia development, our results suggest that pain‐related behaviors were present during the last 4 to 5 experimental days, suggesting that the denervation process had already been initiated in parallel to the development of pain‐related behavior. Importantly, we report the first evidence of denervation in myeloma‐bearing bones, suggesting unstudied mechanisms that require further attention. As systemic administration of the same cell line in BKAL mice did not cause pain‐related behaviors, we suggest that myeloma‐induced bone pain is the result of combinatorial mechanisms including osteolytic lesion development and bone marrow denervation, and that multitarget drugs may be needed for its effective treatment.

Developing optimal preclinical models for the study of disease mechanisms is of primary importance. Here, we present the first in‐depth characterization of an immunocompetent model of myeloma‐induced bone pain, which allows the evaluation of site‐specific pain‐related behavior through the use of non‐stimulus‐evoked behavioral tests. Furthermore, intrafemoral inoculation of myeloma cells induces the development of osteolytic lesions and denervation of the tumor‐bearing bone in this model, suggesting that myeloma‐induced bone pain may be caused by a combination of osteolytic bone lesions and myeloma‐induced nerve damage. We suggest that the intrafemoral 5TGM1 model is a useful tool to further understand the mechanisms of myeloma bone disease and myeloma‐induced bone pain, which is of foremost importance to improve the quality of life of multiple myeloma patients.

## Disclosure

The authors have nothing to disclose.
